# The somatostatinergic system in the mammalian cochlea

**DOI:** 10.1186/1471-2202-12-89

**Published:** 2011-09-06

**Authors:** Vesna Radojevic, Claudia Hanusek, Cristian Setz, Yves Brand, Josef P Kapfhammer, Daniel Bodmer

**Affiliations:** 1 From the Department of Biomedicine University Hospital Basel and the Clinic for Otorhinolaryngology, University Hospital Basel, Petersgraben 4, 4031 Basel, Switzerland; 2 Anatomical Institute, Department of Biomedicine, University of Basel, Pestalozzistr. 20, 4056 Basel, Switzerland

## Abstract

**Background:**

Little is known about expression and function of the somatostatinergic system in the mammalian cochlea. We have previously shown that somatostatin administration may have a protective effect on gentamicin-induced hair cell loss. In this study, we have analyzed the cochlear expression of somatostatin receptor 1 (SST1) and somatostatin receptor 2 (SST2) at both the mRNA and the protein level in wild-type mice, as well as in SST1 and SST2 knock-out (KO) mice and in cultivated neurosensory cells.

**Results:**

We demonstrate that the somatostatin receptors SST1 and SST2 are specifically expressed in outer and inner hair cells (HCs) of the organ of Corti (OC), as well as in defined supporting cells. The expression of SST1 and SST2 receptors in cultivated P5 mouse OC explants was similar to their expression in inner and outer hair cells. Somatostatin itself was not expressed in the mammalian cochlea, suggesting that somatostatin reaches its receptors either through the blood-labyrinthine barrier from the systemic circulation or via the endolymphatic duct from the endolymphatic sac. We used mice with a deletion of either SST1 or SST2 to learn more about the regulation of SST1 and SST2 receptor expression. We demonstrate that in SST1 KO mice, SST2 was expressed in outer HCs and Deiters' cells, but not in pillar cells or inner HCs, as compared with wild-type mice. In contrast, in SST2 KO mice, the expression pattern of the SST1 receptor was not altered relative to wild-type mice.

**Conclusions:**

These findings reveal that somatostatin receptors demonstrate specific expression in HCs and supporting cells of the mouse cochlea, and that absence of SST1 alters the expression of SST2. This specific expression pattern suggests that somatostatin receptors may have important functional roles in the inner ear.

## Background

Somatostatin, also known as somatotropin release-inhibiting factor (SRIF), is mainly produced by endocrine, gastrointestinal, immune, and neuronal cells, as well as by certain tumors. Somatostatin is widely distributed throughout the central nervous system (CNS) and peripheral tissues in mammals [1]. The discovery of somatostatin receptor subtypes triggered in-depth research into their binding properties and their coupling to multiple signaling pathways. Somatostatin acts via a family of G-protein-coupled receptors known as somatostatin receptors 1-5 (SST1 - SST5), which are differentially distributed throughout the CNS [2]. Signaling through somatostatin receptors is complex and involves auto-, para-, or endocrine mechanisms [3-8]. Binding of somatostatin to its receptors induces G-protein activation through various pathways, resulting in the activation of several key enzymes, including adenylyl cyclase, phosphothyrosine phosphatase (PTPase) and mitogen activated protein kinase (MAPK) are modulated, along with changes in the intracellular levels of calcium and potassium ions [9].

Studies over the last few years in mice have shown that somatostatin and its receptors appear to play an important role in cell death. In a retina ischemia model, activation of the SST2 receptor protected retinal neurons from damage [10]. Additionally, studies in mice with genetic alterations of the somatostatinergic system revealed that an increased presence of functional SST2 receptor protected against retinal ischemia [11]. Therefore, SST2 analogues might be of therapeutic benefit in retinal diseases [12-14].

However, in contrast to the situation in the retina, less is known regarding the expression or function of somatostatin and its receptors in the inner ear. Tachibana et al. reported on somatostatin-like immunoreactivity in the medial geniculate body, cochlear nucleus, inferior colliculus, auditory cortex, and cochlea, but did not find somatostatin-like immunoreactivity in the cochlear perilymph [15]. Somatostatin-like immunoreactivity has also been observed in the cochlear nuclei of postnatal rats and it has been suggested that somatostatin might be important for the development of the auditory system [16]. In an additional study, somatostatin-producing cells were observed in the covering epithelium of the spiral prominence and in the epithelium of the intermediate and rugosal part of the endolymphatic sac [17,18]. In a recent publication from our group, we demonstrated expression of SST1 and SST2 mRNA in the postnatal rat cochlea and we reported on a dose-dependent protective effect of somatostatin on gentamicin-induced HC loss *in vitro *[19].

In the present study, we have analyzed the cochlear expression of SST1 and SST2 at both the mRNA and protein level in wild-type mice, as well as in SST1 and SST2 KO mice and in cultivated neurosensory cells.

## Results

### SST1 and SST2 mRNAs are expressed in the cochlea

**Figure 1:**
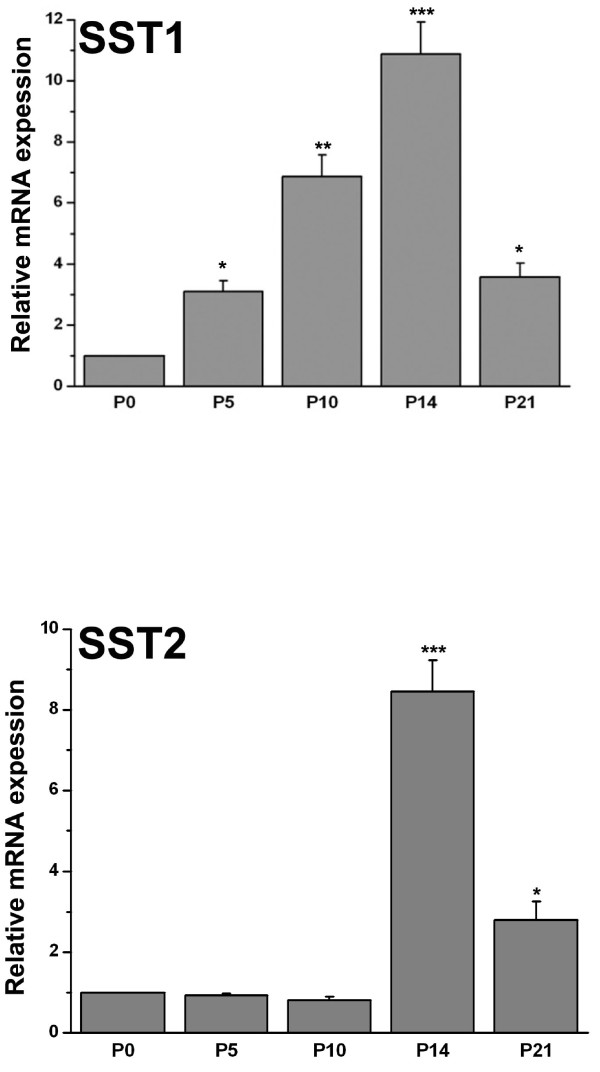
**SST1 and SST2 cochlear gene expression in OC explants, P0-P21**. The relative distribution of SST1and SST2 mRNA expression in OC tissue from wild-type mice of different postnatal ages was quantified by real-time PCR. GAPDH served as an endogenous control. Gene expression levels are expressed as mean (± S.E.) fold increase as compared with values obtained in OC explants from P0 mice. Data were obtained from 5 independent experiments. Statistical analysis was performed using the ANOVA test followed by the Bonferroni post-hoc test. *, p < 0.02; **, p < 0.0002; ***, p < 0.00001.

We performed real-time PCR to determine the quantitative gene expression of SST1 and SST2 in in the OC of 0-, 5-, 10-, 14-, and 21-day-old wild-type mice. Expression of SST1 is significantly increased in OC explants from P5, P10, P14 and P21 day-old-mice compared to the expression in OC explants from P0 mice (Figure 1). As shown in Figure 1, SST2 mRNA appeared to be expressed at a low level in the cochlea of P0, P5, and P10 mice. The expression of SST2 in OC explants from P14 and P21 old wild-type mice is significantly increased as compared to expression in OC explants from P0, P5 and P10 mice.

### SST1 and SST2 expression in wild-type and KO mice

Immunohistochemistry was performed to localize SST1 and SST2 protein in the adult mouse cochlea. Cochlear tissue sections were stained with SST1 and SST2 antibodies, respectively; cell nuclei were stained blue with DAPI. Immunostained sections were analyzed by confocal microscopy.

#### Cochlear SST1 and SST2 protein expression in adult mice

**Figure 2:**
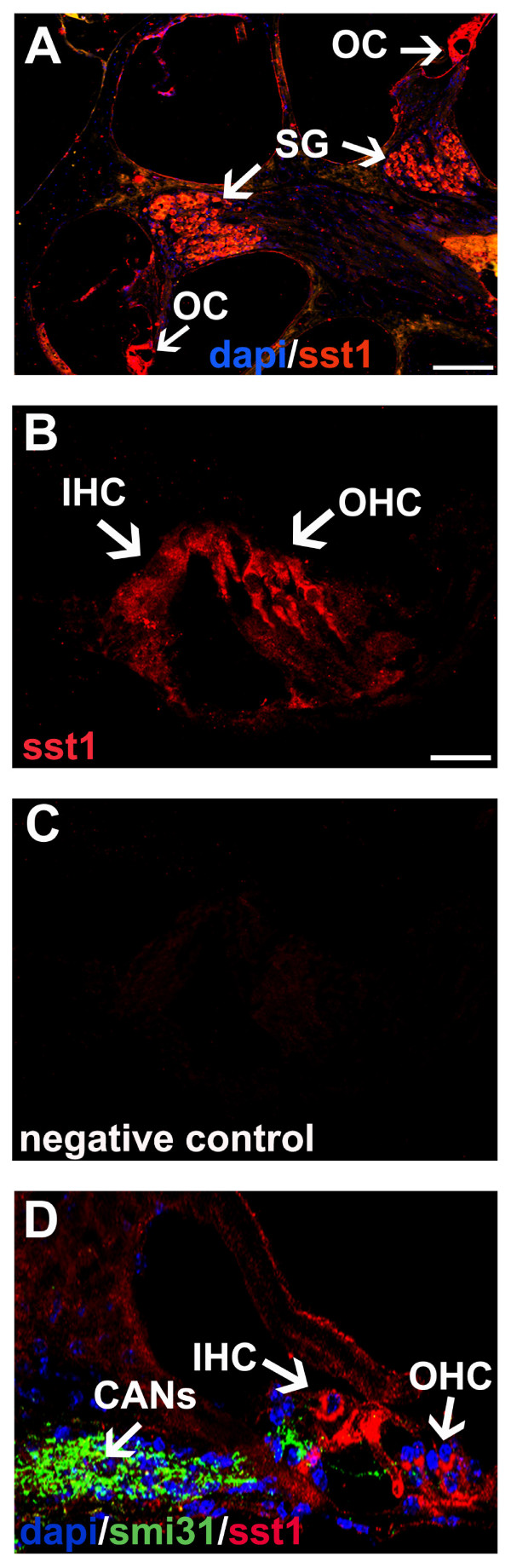
**SST1 in the adult mouse cochlea**. A, overview, SST1 (red) can be observed in the OC and SG; cell nuclei (DAPI) are in blue. B, SST1 (red) can be detected in outer HCs, inner HCs, and outer and inner pillar cells. C, D, labeling with the neurofilament marker SMI31 (green) demonstrates that cochlear afferent neurons (CANs), which connect the SG with the OC, do not express SST1; cell nuclei (DAPI) are in blue. (A) Image by immunofluorescence microscopy, scale bar = 50 μm. (B-D) Images by confocal microscopy, scale bar = 25 μm.

SST1 immunoreactivity was observed in outer and inner HCs, in outer and inner pillar cells, and in the spiral ganglion (SG) (Figure 2A and 2B). The strongest immunoreactivity was observed in the apical part of the outer and inner HCs (Figure 2B), and weaker staining was present in the basal part of the HCs and the pillar cells. Double labeling with the neurofilament marker SMI31, which stains axons, revealed that the axons connecting the SG with the OC do not express SST1 (Figure 2D) SST1 immunostaining was completely absent in the SST1 KO mice, confirming the specificity of the antibodies used (Figure 2C).

**Figure 3:**
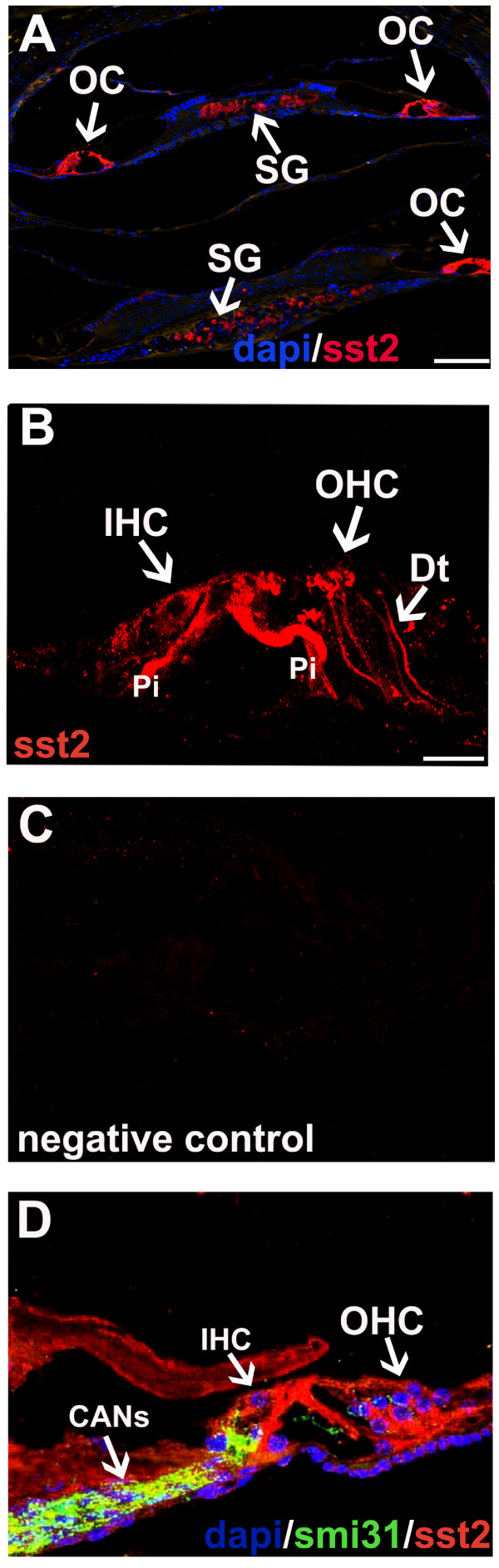
**SST2 in the adult mouse cochlea**. A, overview, SST2 (red) can be observed in the OC and the SG; cell nuclei (DAPI) are in blue. B-C, SST2 (red) can be detected in outer HCs, inner HCs, and the membrane of Deiters' cells, as well as in outer and inner pillar cells. D, labeling with the neurofilament marker SMI31 (green) demonstrates that cochlear afferent neurons (CANs), which connect the SG with the OC, do not express SST2; cell nuclei (DAPI) are in blue. (A) Image by immunofluorescence microscopy, scale bar = 50 μm. (B-D) Images by confocal microscopy, scale bar = 25 μm.

SST2 immunoreactivity was observed in the OC (Figure 3A), namely in the inner and outer HCs, the inner and outer pillar cells, and at the membrane of Deiters' cells (Figure 3B and 3D). Staining intensity appeared to be strongest in the pillar cells and Deiters' cells and less strong in outer and inner HCs. SMI31-positive axons from the spiral ganglion were SST2-negative (Figure 3D). SST2 immunostaining was completely absent in the SST2 KO mice, confirming the specificity of the antibodies used (Figure 3C).

#### Cochlear SST2 protein expression in adult SST1 receptor KO mice

**Figure 4:**
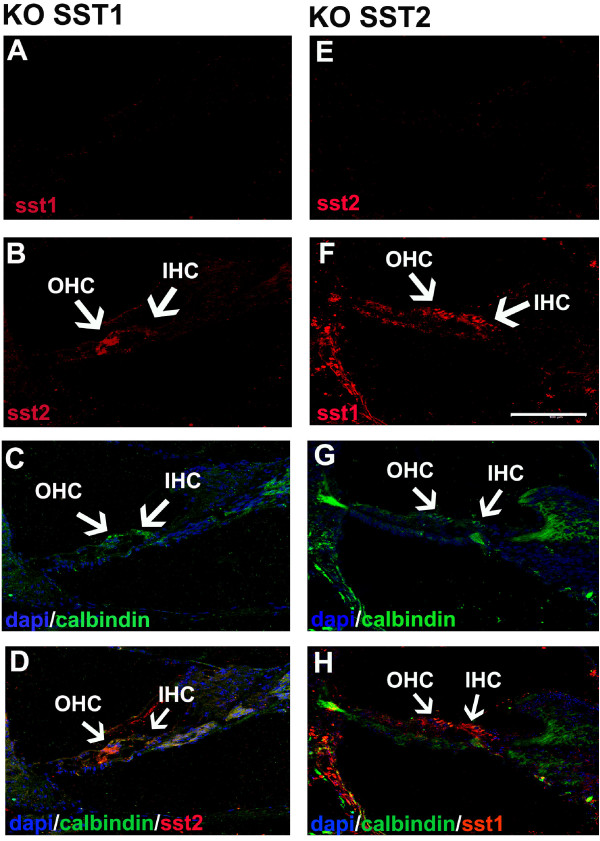
**Cochlear SST1 and SST2 localization in SST1 and SST2 KO mice**. A, E, SST1 (A, red) cannot be detected in the SST1 KO mouse cochlea, and SST2 (E, red) cannot be detected in the SST2 KO mouse cochlea (negative controls). B, F SST2 (B, red) is expressed in outer HCs and Deiters' cells in the SST1 KO cochlea, while SST1 (F, red) is expressed in outer and inner HCs, as well as in supporting cells in the SST2 KO cochlea. C, G, Cell nuclei (DAPI, blue) and calbindin staining (green) in cochleae from SST1 KO (C) and SST2 KO (G) mice. D, H, Composite images of cochleae from SST1 KO (D) and SST2 KO (H) mice. Images by confocal microscopy; scale bar = 100 μm.

No SST1 antibody staining was observed in the cochlea of SST1 KO mice, indicating the complete absence of SST1 protein in this mouse model and confirming the specificity of the antibody used (Figure 4A). SST2 protein (Figure 4B and 4D) was strongly expressed in Deiters' cells, and to a lesser extent in outer HCs, but was absent from pillar cells and inner HCs compared with wild-type mice (Figure 3B). This expression pattern of SST2 in SST1 receptor KO mice was observed in all of the sections analyzed (n = 20 sections analyzed per KO mouse).

#### Cochlear SST1 protein expression in adult SST2 receptor KO mice

No SST2 antibody staining was observed in the cochlea of SST2 KO mice, indicating the complete absence of SST2 protein in this mouse model and confirming the specificity of the antibody used (Figure 4E). SST1 protein (Figure 4F and 4H) was expressed in outer and inner HCs, as well as in supporting cells. The overall staining pattern did not differ from that of wild type mice (compare Figure 2B).

### SST1 and SST2 protein are expressed in OC explants

**Figure 5:**
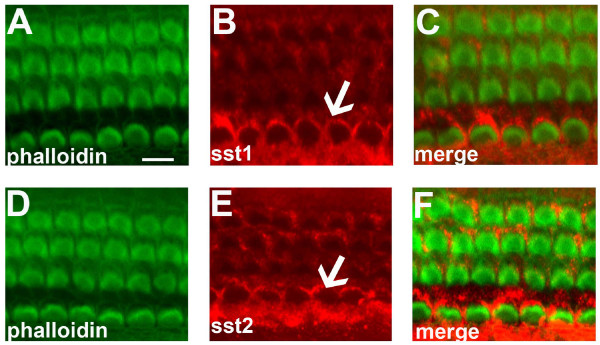
**SST1 and SST2 expression in OC explants**. A, D, phalloiding staining (green) of OC explants. B, E, immunoreactivity of both SST1 and SST2 (red) was stronger in inner than in outer HCs of OC (arrow). C, F, Respective combined images of phalloidin (green) with SST1 and SST2 (red) confirm that the inner and outer HCs in cultivated OC express SST1 and SST2. Images by immunofluorescence microscopy; scale bar = 50 μm.

To determine whether SST1 and SST2 were expressed in OC explants prepared from postnatal mice, we double-stained OC explant tissue with phalloidin and either SST1 or SST2 antibodies (Figure 5). Phalloidin staining reveals that the cytoarchitectonic organization of the OC is maintained, and inner HCs and the three outer layers of HCs can be identified (Figure 5A and 5D). Both SST1 and SST2 were most strongly expressed in inner HCs; however, weak immunoreactivity was also present in the outer HCs (Figure 5B, C, E, and 5F).

### SST1 and SST2 protein expression in passaged neurosensory cells derived from the cochlea

**Figure 6:**
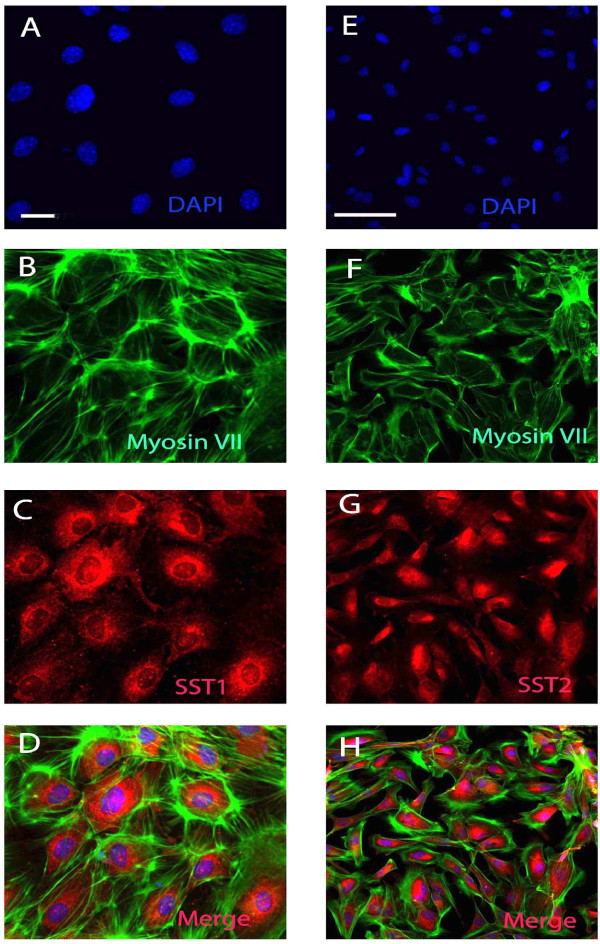
**Detection of SST1 and SST2 in passaged neurosensory cells from mouse cochleae**. A, E, cell nuclei (DAPI) are depicted in blue. B, F, the cell membrane of the cultivated cells is positive for the HC marker myosin VII (green). C, G, perinuclear localization of SST1 (C, red) and SST2 (G, red) are detected in cultivated cells. D, H, composite images. Images by immunofluorescence microscopy; scale bar = 50 μm.

Immunocytochemistry was performed to localize SST1 and SST2 protein on passaged neurosensory cells from the immature cochlea. Myosin VII served as a marker for HCs, and cell nuclei were stained in blue with DAPI. SST1 and SST2 were detected in the Myosin VII-positive neurosensory cells and were mainly localized in the perinuclear region (Figure 6C, D, G, and 6H). Labeling of the plasma membrane was weak or undetectable in the cultured cells, suggesting that most receptors were not inserted into the plasma membrane.

### Cochlear expression of somatostatin

**Figure 7:**
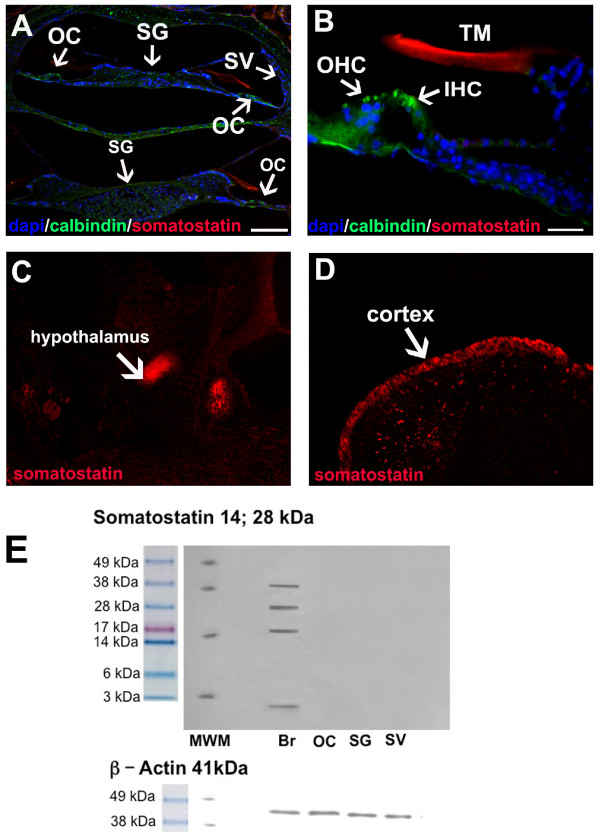
**Detection of somatostatin in the wild-type mammalian cochlea**. A, B, somatostatin (red) cannot be detected in the cochlea of WT animals, although there is positive somatostatin staining on the tectorial membrane (B, TM); cell nuclei (DAPI) are in blue, and calbindin staining is in green. C, D, somatostatin-positive staining (red) is evident in the cortex and hypothalamus of WT mouse brain. E, Western blotting reveals that somatostatin can only be detected in protein extracts from brain tissue (positive control) but not in extracts from the OC, SV or SG. Actin serves as a standard to demonstrate that equal amounts of proteins have been loaded. Images by immunofluorescence microscopy; scale bar = 50 μm (A, C) or 25 μm (B, D).

Immunohistochemistry was performed to localize somatostatin in the adult mouse cochlea. Cochlear tissue sections were stained with somatostatin antibodies. No somatostatin immunoreactivity was observed in the cells of the cochlea (Figure 7A and 7B). The label in the tectorial membrane is likely to reflect unspecific staining. Somatostatin was detected in the brain via Western blotting (positive control) (Figure 7E). In the brain sample, we observed bands at 1,600 Da, 17,000 Da (corresponding to somatostatin 14), 28,000 Da, and 38, 000 Da (corresponding to somatostatin 28). We were unable to detect somatostatin in protein extracts from the OC, SG, or SV. These results suggest that somatostatin is not produced in the mammalian cochlea.

## Discussion

In the present study, we demonstrate that the somatostatin receptors SST1 and SST2 are expressed in outer and inner HCs of the OC, as well as in defined supporting cells. We also demonstrate that in SST1 KO mice, SST2 is strongly expressed in Deiters' cells and outer HCs, but not in pillar cells or inner HCs compared with wild-type mice. In contrast, in SST2 KO mice, the expression pattern of SST1 receptor is not altered compared with wild-type mice. Finally, we provide evidence that somatostatin is not produced in the cochlea itself.

### Localization of SST1 and SST2 receptors in the inner ear

At present, very little is known about the expression of SST1 and SST2 receptors in the inner ear. Our findings demonstrate that SST1 and SST2 receptor mRNA is expressed in the mammalian cochlea. Furthermore, we were able to localize the expression of both receptors in the cochlea using immunohistochemistry. We show that SST1 and SST2 receptors are present specifically in HCs, as well as in supporting cells of the OC of the adult mouse, with SST1 being more prominent in HCs and SST2 being more prominent in supporting cells. Both receptors were present in the SG. It is noteworthy that SST1 and SST2 m RNA expression increases postnatal, and peaks at P14. Brain stem electrical response audiometry (BERA) cannot be recorded before P12-P14 in mice. It might be possible that SST1 and SST2 are necessary for the growth and development of the OC but not for later maturation of hearing. The SST1 and SST2 receptor proteins are also expressed in passaged cochlear neurosensory cells derived from postnatal mouse OC. In the future, *in vitro *experiments such as calcium influx assays would be useful to test somatostatin function.

### Neuroprotective role of SST1 and SST2 receptors in the inner ear

The function of these two receptors in the OC is currently unknown. However, we have recently demonstrated that somatostatin can protect HCs from aminoglycoside toxicity in a dose-dependent manner *in vitro *[19]. It is reasonable to assume that this neuroprotective effect of somatostatin on HCs was mediated by SST1 or SST2 receptors; however, this has not yet been experimentally proven. Nevertheless, our findings are consistent with a neuroprotective role for the somatostatin signaling system with respect to auditory HCs.

In contrast to the situation in the inner ear, more is known about the expression and neuroprotective role of the somatostatinergic system in the retina. It has been demonstrated that somatostatin and its receptors (SST1-SST5) are expressed in the retina, predominatly in amacrine cells and bipolar cells [14,20]. Moreover, activation of the somatostatin receptor SST2 by somatostatin or its analogues has been shown to protect retinal neurons against ischemia-induced damage [10]. In addition, studies in mice with genetic alterations of the somatostatinergic system have revealed that an increased presence of functional somatostatin receptor SST2 protects against retinal ischemia [11]. Therefore, SST2 analogues might be of therapeutic benefit in retinal diseases such as glaucoma or diabetic retinopathy, but may also protect from hearing loss due to HC degeneration and death.

What are the molecular mechanisms involved in the protection of cells from death by somatostatin and its analogues? Studies in mouse retinal explants have demonstrated that the SST2 receptor inhibits potassium-induced glutamate release [21]. By limiting the amount of glutamate available to glutamate receptors, somatostatin and its analogues may exert a neuroprotective function against glutamate neurotoxicity, which characterizes many retinal diseases [1]. Glutamate excitotoxicity appears to be mediated by the activation of caspase-3, as shown in cerebrocortical neurons [22]. Glutamate excitotoxicity is also involved in HC damage and death in the cochlea [23]. Therefore, somatostatin may protect HCs from aminoglycoside toxicity, either by limiting glutamate release or by mitigating the toxic action of excess glutamate on HCs. In this context, it is notable that the somatostatin analogue octreotide alters the activity of the phosphatidylinositol 3-kinase pathway [24] in pituitary tumor cells. We demonstrated recently that the phosphatidylinositol 3-kinase pathway is involved in NF-kappaB-dependent HC survival [25]. Therefore, it might be possible that somatostatin exerts its effect on HCs through the phosphatidylinositol 3-kinase survival pathway.

### Absence of somatostatin in the cochlea

Using immunohistochemistry and Western blotting, we were unable to detect somatostatin within the mammalian cochlea. Because we show the expression of somatostatin receptors within the cochlea, the following question arises: how can somatostatin reach its receptors? One possibility would be that somatostatin production is induced only upon excitotoxic challenge or other adverse signals. However, somatostatin immunoreactivity has been observed in the endolymphatic sac [18]. Therefore, it might be possible that somatostatin is produced in the endolymphatic sac and reaches the cochlea through the endolymphatic duct. Another possibility might be that somatostatin crosses the blood-labyrinthine barrier, and so somatostatin within the blood could reach the cochlea. It has been demonstrated that octapeptide analogs of somatostatin can cross the blood-brain barrier via a saturable transport system [26]. A similar transport system might be responsible for the transport of somatostatin into the cochlea via the blood-labyrinthine barrier.

### SST1 KO mice: loss of SST1 receptors

In the inner ear, there is no information available on the consequences of SST1 receptor deletion in mice. In the cochlea of SST1 KO mice, SST1 protein was completely absent. This result is consistent with the SST1 gene being completely inactivated [27], and confirms the specificity of the antibody used. Our data demonstrate that in the mouse cochlea, SST1 receptor loss has a major effect on the expression of SST2 receptors: while in wild-type mice SST2 could be observed in outer HCs, inner HCs, and supporting cells (and most prominently in outer and inner pillar cells), in SST1 KO mice the SST2 receptor was observed only in outer HCs and cells supporting the outer HCs, but not in pillar cells or inner HCs. Notably, a similar observation has been made in the mouse retina: SST1 receptor loss resulted in a pronounced increase of SST2 receptor expression [21]. Among other possibilities, the authors of this study speculated that the SST1 receptor might directly regulate SST2 receptor expression. Our findings demonstrate prominent compensatory regulation in the mammalian cochlea as a consequence of a distinct somatostatin receptor deletion. This compensatory mechanism is subtype-specific, as it is observed only after the deletion of SST1, and not after the deletion of SST2.

### SST2 KO mice: loss of SST2 receptors

SST2 protein was undetectable in the cochlea of SST2 KO mice. This result is in agreement with the complete inactivation of the responsible gene [27], and also confirms the specificity of the antibody used. In contrast to SST1 KO mice, SST2 receptor loss has no effect on the expression of the SST1 receptor in the inner ear. In wild-type mice as well as in SST2 KO mice, the SST1 receptor was detected in outer and inner HCs, as well as in supporting cells. This finding is in agreement with observations in other cellular systems where no such compensatory effect was observed following the knockout of a somatostatin receptor. For example, in the brain of SST2 KO mice, little effect has been observed on the expression of SST1 and SST3-5 [28].

## Conclusions

The presence of somatostatin receptors within the mammalian cochlea, their specific expression in the OC, and their subtype-specific compensatory regulation as a consequence of distinct somatostatin receptor deletion suggest an important role for the somatostatinergic system within the inner ear.

## Methods

### Animals

Experiments were performed on mouse cochleae from wild-type (WT) C57BL/6 mice of both sexes, in the adult (n = 5) and during postnatal (n = 10/postnatal day) development. Immunohistochemstry was also performed on cochleae from adult SST1 (n = 4) or SST2 (n = 4) KO strains of both sexes. An SST1 null allele was generated by deleting the entire SST1 coding region, targeted and maintained in a hybrid129/Sv: C57BL/6 background [29]. The WT and homozygous SST2 KO mice [27,30] were backcrossed into the C57BL/6J genetic background for thirteen generations.

All animal procedures were conducted in conformity with the European Communities Council Directive of 24 November 1986 (86/609/EEC) and were reviewed and permitted by the Kantonales Veterinäramt, Basel, Switzerland.

### Dissociation and cultivation of HCs

The cochleae of 6-day-old mice were dissected to isolate the OC [31]. The OC was placed in preparation medium (PM) consisting of DMEM (Gibco-Invitrogen, Switzerland) with 25 mM HEPES (Gibco-Invitrogen, Switzerland), 6 mg/ml glucose, 30 U/ml penicillin, and 30 ml/ml N-2 supplement (Cell Concepts, Switzerland) at pH 7.3. The tissue was cut into 2-mm cubes and transferred to a sterile 15-ml tube. Dissected tissue pieces were rinsed twice in PM and trypsinized for 15-20 min at 37°C. Trypsinization was stopped by the addition of horse serum (one-quarter volume) and DNase (0.01%). The cells were then centrifuged for 5 min at 600 g at room temperature. The pellet containing 100,000 cells/ml was resuspended, and cells were plated onto poly-L-lysine-coated cultured dishes for 7 days. Culture dishes were coated with 50-100 μL of 10 μg/mL poly-D-lysine, and pretreated with 10% heat-inactivated FCS for 2 hours. The cells were seeded at a density of 0.5-1.0 × 10^6 ^cells/cm^2 ^and incubated in complete growth medium, which consisted of PM and the brain-derived neurotrophic factor (10 ng/mL). Trypan blue exclusion indicated that this preparation consisted of 87-98% viable cells.

For passaging, cells were dissociated mechanically and were resuspended in the same medium at a density of 50,000 cells/ml. The cells were then passaged 10 times and subsequently analyzed by immunocytochemistry.

### OC explant cultures

OC explants were prepared and cultivated according to as detailed by Sobkowicz et al. [32]. Neonatal (P5, n = 20) wild-type mouse pups were rapidly decapitated. After removing the brains and the temporal bones, the cochleae were microdissected, and the OC, SG, and SV were isolated. After isolation, all OCs were separated, placed on Millicell-CM 0.4-μm culture plates (Millipore, Austria) in 24-well dishes, and were maintained in culture medium at 37°C in a humidified CO_2_-enriched atmosphere for a minimum of three days to allow tissue recovery from explantation trauma. The medium consisted of DMEM with 25 mol/l HEPES, supplemented with 10% fetal calf serum (Invitrogen, Carlsbad, CA), and 30 U/ml penicillin (Sigma) adjusted to pH 7.3.

### RNA extraction

Twenty OCs from wild-type mouse pups aged P0-P21 were placed separately in RNAlater (Qiagen, Hombrechtikon, Switzerland). RNA isolation of the brains and the inner ear components were performed using the RNAeasy Minikit (Qiagen) and employing a Ultra-Turrax T8 tissue homogenizer (IKA-Werke, Staufen, Germany) according to the manufacturer's instructions, including DNAse treatment. The quantity and quality of isolated RNA were determined with NanoDrop ND 1000 (NanoDrop Technologies, Delaware, USA). The 260/280-nm ratio of all samples was between 1.8 and 2.1

### Real-time PCR

Total RNA (500 ng) was reverse transcribed into cDNA with the First Strand cDNA synthesis kit (Roche Applied Biosciences) according to the manufacturer's instructions. The reaction took place in an ABI Prism 7900HT Sequence Detection System (Applied Biosystems) using a Fast Start Universal SYBR Green Master (Rox) (Roche Applied Biosciences Foster City, USA). The primer sequences were:

SST1-forward, 5'-CAGGTTTAAAGAACTGGCAAGC-3',

SST1-reverse 5'-ATTAATAAGCGGCACCATCG-3',

SST2-forward5'-TCTTTGCTTGGTCAAGGTGA-3',

SST2-reverse 5'-TCCTGCTTACTGTCGCTCCT -3' (Microsynth, St. Gallen, Switzerland). Each reaction contained 300 nM of primer. The cycling parameters were 10 min at 95°C, followed by 40 cycles of 15 s at 95°C and 60 s at 60°C. We calculated relative quantities of specifically amplified cDNA with the comparative threshold cycle method. GAPDH acted as an endogenous reference (Microsynth). Template-free and reverse-transcription-free controls ensured that nonspecific amplification and DNA contamination could be excluded.

### Preparation of paraffin cochlear sections

Mice were killed with an overdose of sodium pentobarbital (100 mg/kg) and transcardially perfused with 50 ml of phosphate-buffered 4% paraformaldehyde (pH 7.4, at 4°C). The inner ear was carefully removed. Decalcification was carried out in a light-protected flask for 10 days in a solution of 120 mM EDTA (Merck, New Jersey, USA) in distilled water (pH 6.8). After decalcification, cochleae were prepared for paraffin embedding. Briefly, cochleae were dehydrated in graded ethanol solutions (at 70%, 80%, 95%, and 3 × 100% each for 1 h; 3 × xylol for 1 h; 2 × paraplast at -60°C for 1 h; and paraplast at -60°C for 10 h), and embedded in paraffin at 56°C. After perfusion, the brain was removed from the skull and post-fixed overnight.

### Histology and Immunocytochemistry

For histological evaluation, cochlear sections of 10 μm thickness were cut on a Leitz microtome and mounted on Superfrost plus slides (Menzel, Braunschweig, Germany). Sections were deparaffinized, rehydrated, and then underwent antigen unmasking by boiling in 10 mM sodium citrate buffer (pH 6.0) and maintenance at sub-boiling temperature for 10 min. The slides were then cooled and washed in PBS for 5 min and proceeded to immunohistochemistry. For histological evaluation of the brain, coronal sections of 30 mm thickness were collected and mounted on Superfrost plus slides (Menzel, Germany) and dried at room temperature.

Cells attached to poly-lysine-coated cover slips were fixed in 4% paraformaldehyde for 15 min, washed twice, and kept in 0.01 M phosphate-buffered saline (PBS) at 4°C for further processing. After fixation and permeabilization in PBS containing 0.5% Triton X-100 (PBS-T, pH 8), the vibratome brain sections and cell cultures were incubated for 1 h at room temperature in blocking solution containing PBS-T and 3% normal goat serum (NGS). Microtome sections of mouse cochlea were incubated for 1 h at room temperature in blocking solution PBS-T containing 5% Triton X-100 (pH 8) and 3% NGS. The sections and cell culture were incubated with primary antibody diluted in PBS-T with 1% NGS overnight at 4°C. The following primary antibodies were used: rabbit polyclonal anti-SST1 and anti-SST2 antibodies (1:400, Gramch Laboratories, Germany), rabbit polyclonal anti-VIIa antibody (1:500, Abcam, UK), mouse monoclonal anti-calbindin antibody (1:500, Chemicon, Billerica, USA), mouse monoclonal anti-SMI31 antibodies (1:250, Chemicon, Billerica, USA), and monoclonal anti-somatostatin antibody (1:250, Santa Cruz Biotech, California, USA). After 3 washes in PBS-T, the sections were incubated for 1 h at room temperature with the appropriate secondary antibodies (1:250, Alexa-conjugated, Molecular Probes, Lubio Science, Switzerland) diluted in PBS-T with 1% NGS for 2 h at room temperature. After washing in PBS, the sections were counterstained with DAPI and mounted on glass slides with Mowiol.

Slices were visualized on an Olympus AX-70 microscope equipped with a spot digital camera. Recorded images were adjusted for brightness and contrast with Image-Pro Plus and Photoshop image processing software.

### Western Blotting

Animals were decapitated and 20 cochleae were carefully micro-dissected in ice-cold PBS. The OC was separated from the SG and the SV. Brain extract was used as a positive control. Explants were homogenized in CelLytic buffer containing protease inhibitor cocktail (Sigma-Aldrich, St. Louis, USA), and centrifuged. Supernatants were aspirated and placed in a new tube. Protein concentration was determined using the BCA Protein Assay Reagent kit (Pierce, Rockford, USA) according to the manufacturer's instructions. Lysates were mixed with Novex Tricine SDS sample buffer X2 (Invitrogen, Switzerland) and heated at 95°C for 5 min. Fifty micrograms of each lysate was resolved on a Novex Tricine 10% gel; 5 μl of SeeBlue Plus2 Standard (Invitrogen, Switzerland) were used.

Samples were run at 30 V constant in Novex Tricine Running Buffer (Invitrogen, Switzerland) until samples had completely run through the stacking gel and at 100 V constant until the ion front reached the bottom of the gel (approximately 2 h). The sampels from the tricine gel were transferred to a polyvinylidene fluoride membrane (Immobilion -P^SQ ^Millipore, Switzerland). The transfer was conducted in Novex Tricine Transfer Buffer (Invitrogen, Switzerland) at 4°C for 2 h at 400 mA constant for 2 gels. The non-specific sites of the transferred proteins were blocked with blocking solution (1.54 mM KH_2_PO4; 155.17 mM NaCl; 2.71 mM Na_2_HPO4-7H_2_O, pH 7.2; 0.1% Tween-20; Roche, Switzerland) diluted (1:100) in PBS-T for 1 h at room temperature. The membrane was washed with PBS-T (3 × 10 min) and then incubated with primary antibodies in 5% non-fat dry milk in PBS. The following primary antibodies were used: mouse monoclonal anti-somatostatin (1:1000, Abcam, UK) and mouse monoclonal anti-β-actin (1:2000, Santa Cruz Biotech, California, USA). The membranes were incubated with the primary antibodies overnight at 4°C.

The blots were washed with PBS-T (3 × 10 min) and incubated with appropriate HRP-conjugated secondary antibody for 1 h at room temperature. After washing, bands were visualized using enhanced chemiluminescence (ECL; Amersham Biosciences, Piscataway, USA). Serial exposures were made to radiographic film (Hyperfilm ECL; Amersham Biosciences).

### Statistical Analysis and Software

Since more than two samples were compared, analysis was performed by the ANOVA test followed by the Bonferroni post-hoc test. The Origin software (Microcal Software, Inc., Northampton, MA) was used to generate graphs and for statistical analysis.

## Authors' contributions

VR conceived the study, designed the experiments, analysed the findings, and wrote the manuscript; CH, CS, and YB carried out the experiments; JPK helped to draft the manuscript; DB designed the study and help to draft the manuscript. All authors read and approved the final manuscript.
